# Adherent Peanut Image Segmentation Based on Multi-Modal Fusion

**DOI:** 10.3390/s24144434

**Published:** 2024-07-09

**Authors:** Yujing Wang, Fang Ye, Jiusun Zeng, Jinhui Cai, Wangsen Huang

**Affiliations:** 1 College of Metrology Measurement and Instrument, China Jiliang University, Hangzhou 310018, China; rebecca.wyj@foxmail.com (Y.W.); caijinhui@cjlu.edu.cn (J.C.); 2 School of Mathematics, Hangzhou Normal University, Hangzhou 311121, China; jszeng@hznu.edu.cn; 3 Wenzhou Quality and Technology Testing Research Institute, Wenzhou 325052, China; huangwangsen@sina.com

**Keywords:** peanut, adhesion segmentation, line structured light

## Abstract

Aiming at the problem of the difficult segmentation of adherent images due to the not fully convex shape of peanut pods, their complex surface texture, and their diverse structures, a multimodal fusion algorithm is proposed to achieve a 2D segmentation of adherent peanut images with the assistance of 3D point clouds. Firstly, the point cloud of a running peanut is captured line by line using a line structured light imaging system, and its three-dimensional shape is obtained through splicing and combining it with a local surface-fitting algorithm to calculate a normal vector and curvature. Seed points are selected based on the principle of minimum curvature, and neighboring points are searched using the KD-Tree algorithm. The point cloud is filtered and segmented according to the normal angle and the curvature threshold until achieving the completion of the point cloud segmentation of the individual peanut, and then the two-dimensional contour of the individual peanut model is extracted by using the rolling method. The search template is established, multiscale feature matching is implemented on the adherent image to achieve the region localization, and finally, the segmentation region is optimized by an opening operation. The experimental results show that the algorithm improves the segmentation accuracy, and the segmentation accuracy reaches 96.8%.

## 1. Introduction

As a crop with extensive nutritional and application value, peanuts occupy an indispensable and important position in China‘s agricultural system. They are one of the few export-oriented agricultural products with strong international competitiveness. China is the world‘s largest peanut producer, accounting for approximately 40% of the world‘s total peanut output [1,2]. With the rapid development of intelligent agricultural technology, post-production processing has become the key to improving agricultural efficiency and competitiveness. Machine vision [3,4,5,6] can be applied to the automatic identification of weeds and pests as well as to quality inspections, classification, and grading for various agricultural products. As a key step in the breeding of improved varieties of agricultural products, quality detection and sorting directly affect the overall quality of different grades of crops. Therefore, quality in the sorting of peanuts before planting and post-harvest processing [7] is one of the key technologies for ensuring storage quality and improving a product’s grade and added value. However, peanuts are prone to physical damage, insect damage, germination, mildew, and other problems during growth, transportation, and storage, resulting in the uneven quality of peanuts [1]. Therefore, it is of great significance to realize the consistency of peanut-pod quality through quality detection for product reprocessing, product grade improvements, and market competitiveness. On the classification test bench, due to the possible contact and adhesion between peanuts, if collected images are not segmented and preprocessed, this will affect the subsequent peanut quality evaluation [8].

**Figure 1:**
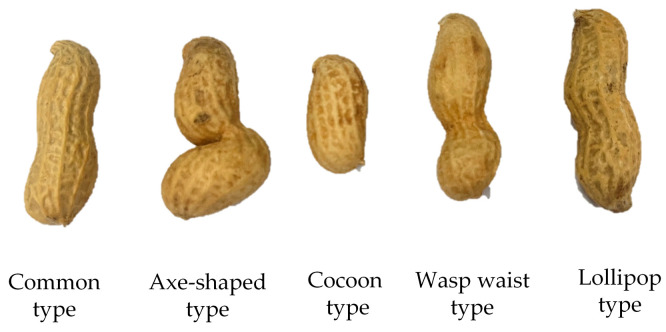
The different shapes of peanut pods.

For image sticking problems, Nee et al. [9] proposed a segmentation algorithm based on a combination of morphology and watershed. Song et al. [10] proposed a segmentation algorithm that combined concave point detection and improved watershed. Wang et al. [11] proposed a new method for obtaining adhesion points based on the curvature direction characteristics of each pixel on the inner and outer contours of adhered rice grains, and they matched the adhesion points according to the curvature extension directions of the adhesion points and the distances between the adhesion points to realize the segmentation of the adhered rice grains. The edge Information of the rice grains was retained as much as possible, and the segmentation accuracy was high. Yan et al. [12] used the Otsu method to preprocess oat grains, and the watershed method was used to segment the adhesion area, which eliminated excessive segmentation, resulting in an accuracy rate that reached 98.55%. However, with increases in the number of oats and overlapping areas, the accuracy of this method was greatly reduced, and so it is only suitable for cases where adhesion is not serious. Jia et al. [13] combined residual network (ResNet) with densely connected convolutional network (DenseNet) to obtain an area where an apple was located with an accuracy rate of 97.31%. Yang et al. [14] proposed a new synthetic image generation and enhancement method to fine-tune the Mask R-CNN network model. The weight loss of the mask was set to two, and the other weight losses were set to one. A loss probability of 0.5 was added to the full connection layer to realize the segmentation of soybean particles and then shape the phenotypic data of a soybean. Based on the research by Araújo [15], Belan et al. [16] proposed a segmentation algorithm that could locate and analyze image particles in space for the quality detection of beans, which further improved segmentation speed and robustness. In offline and online experiments, the segmentation accuracies were 99.6% and 98.5%, respectively, and the processing times were 1.2 s and 1.5 s, respectively. These algorithms have realized the image segmentation of rice, potatoes, wolfberries, and other round targets [17]. However, the surface texture of a peanut pod shell is complex, with the characteristics of a fruit waist and non-uniformity [18], and the fruit’s shapes are diverse [19]. The main types are shown in Figure 1, such as the common type, axe-shaped type, cocoon type, wasp-waist type, and lollipop type. Most of them do not have a round structure, and the traditional segmentation algorithm has had a poor segmentation effect.

In this paper, we propose a multimodal fusion-based image segmentation algorithm for adherent peanut images, which utilizes a line structured light 3D imaging technique to obtain the three-dimensional shape of a peanut and segment it. Then, according to the extraction of the two-dimensional contour of each segmented peanut model as a matching search template, the two-dimensional adherent image segmentation is achieved.

## 2. Principle of 3D-Assisted Segmentation Algorithm

**Figure 2:**
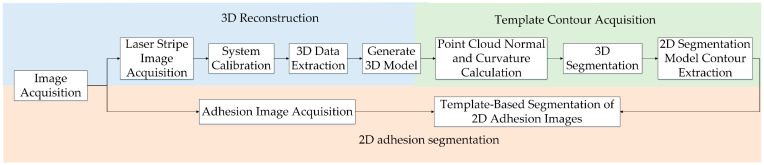
The multimodal fusion segmentation algorithm flow.

Considering that peanut pods typically have irregular shapes and uneven surfaces, and that adhesion usually manifests as a tight fit between peanuts, it is not easy to distinguish 2D peanut images based solely on color or 2D structure. Therefore, a multimodal fusion-based image segmentation algorithm for adherent peanut images is proposed. The flow of the algorithm is shown in Figure 2.

The image segmentation algorithm based on multimode fusion first uses the line structured light to capture the three-dimensional spatial distribution of the adherent peanuts. Subsequently, it employs the three-dimensional morphological edges as a constraint to achieve the segmentation of the two-dimensional adhesion image. Finally, it extracts information on the peanut surface color, texture, and other characteristics.

## 3. Peanut 3D Reconstruction System

### 3.1. Peanut 3D Reconstruction System Architecture

**Figure 3:**
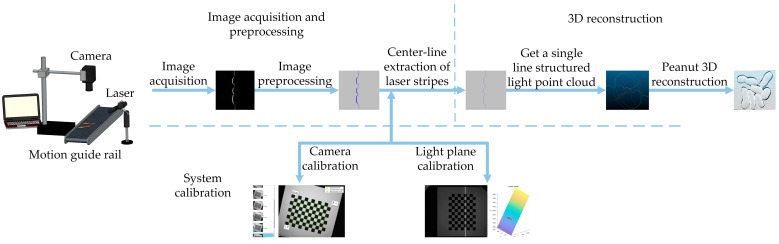
The peanut 3D reconstruction flow.

The system for reconstructing peanuts in three dimensions consists of a laser, an industrial camera, a one-dimensional motion guide, and a computer. The process is shown in Figure 3, which mainly includes three parts: system calibration, image acquisition and preprocessing, and 3D reconstruction. System calibration [20], comprising camera and optical plane calibration, determines the internal and external camera parameters and the optical plane equation parameters [21]. Image acquisition and preprocessing include image acquisition, preprocessing, and laser stripe centerline extraction. Three-dimensional reconstruction is mainly based on the parameters obtained from the previous system calibration, and the three-dimensional coordinates of each independent laser stripe are computed. Simultaneously, combining with the displacement distance of the motion platform, the measured peanut stripe lines are spliced, and the peanut three-dimensional point cloud data are finally obtained.

### 3.2. Principles of Line Structured Light Imaging System

**Figure 4:**
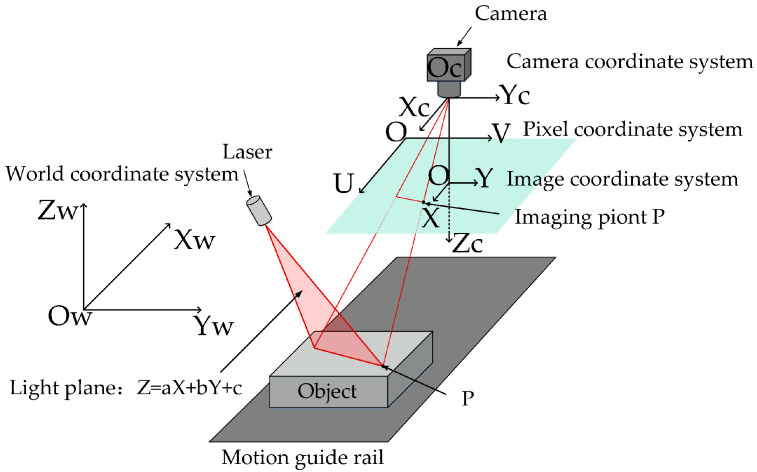
Line structured light 3D schematic.

The line structured optical imaging system with a one-dimensional motion platform is based on the principle of triangulation to obtain surface depth information in the form of a point cloud. The basic structure is shown in Figure 4. A peanut is placed on the motion guide, which moves the peanut unidirectionally for scanning. A laser mounted on top of the guide generates a light plane in space. This light plane intersects with the peanut’s surface, creating a laser stripe [22]. An industrial camera is placed vertically above the guide to capture the laser stripe image. The camera is programmed to take consecutive shots automatically based on the guide’s speed. The system extracts the laser fringe center [23] and spatial mapping geometric relationship [24] to obtain three-dimensional point cloud coordinates of the object’s surface.

The point $O_{c}$ is the optical center of the industrial camera, and the camera coordinate system $O_{c}-X_{c}Y_{c}Z_{c}$ is established with this point as the origin, while the coordinate system O−UV and the coordinate system O−XY are the image pixel coordinate system and the image physical coordinate system, respectively. According to the basic model principle of camera imaging, any point p on the laser stripe can be matched with its corresponding imaging point P′ on the imaging plane. The relationship between the point P′(u,v) in the pixel coordinate system and the point $p(X_{c},Y_{c},Z_{c})$ in the camera coordinate system is as follows:

(1) $$Z_{c}\left(\begin{array}{c} u\\ v\\ 1 \end{array}\right)=\left(\begin{array}{ccc} f_{x} & 0 & u_{0}\\ 0 & f_{y} & v_{0}\\ 0 & 0 & 1 \end{array}\right)\left(\begin{array}{c} X_{c}\\ Y_{c}\\ Z_{c} \end{array}\right)$$

where $f_{x}$ and $f_{y}$ are obtained by dividing the focal length of the camera by the physical dimensions of the pixel point in the X and Y axes of the image physical coordinate system, respectively; $u_{0}$ and $v_{0}$ are the pixel coordinates of the origin O of the image physical coordinate system. Since the feature point p is in the optical plane at the same time, its 3D coordinates should satisfy the optical plane equation. The parameters of the optical plane equation can be obtained through optical plane calibration. Therefore, the feature point $p(X_{c},Y_{c},Z_{c})$ under the camera coordinate system satisfies the following equation:

(2) $$aX_{c}+bY_{c}+cZ_{c}+d=0$$

where a,b,c,d are the parameters of the optical plane equation.

And the coordinates of the feature point p in the world coordinate system $\left(X_{w},Y_{w},Z_{w}\right)$ can be calculated based on the variation relationship between the world coordinate system and the camera coordinate system, that is,

(3) $$\left(\begin{array}{c} \begin{array}{c} X_{w}\\ Y_{w}\\ Z_{w} \end{array}\\ 1 \end{array}\right)={\left(\begin{array}{cc} R & T\\ 0^{T} & 1 \end{array}\right)}^{-1}\left(\begin{array}{c} \begin{array}{c} X_{c}\\ Y_{c}\\ Z_{c} \end{array}\\ 1 \end{array}\right)$$

where R is the rotation matrix and T is the translation matrix, which are also known as the external parameters of the camera and are determined through calibration.

Based on the above principle, the 3D point cloud coordinates of the feature points on the whole laser stripe in the world coordinate system can be determined, thereby completing the 3D reconstruction of a frame of line structured light image.

Given that the camera and laser remain stationary in the system, the peanut motion parameters can be used to implement the corresponding displacement correction and alignment processing for each frame of the acquired data to achieve a complete 3D reconstruction. Since there is a direct relationship between the image captured by the camera and the geometry of the laser projected onto the object’s surface, this study assumes that the physical distances in real life are equivalent to the distances in three-dimensional space. The specific displacements d are formulated as follows:

(4) d=vt

where v is the speed of the peanut’s motion in units of mm/s, and t is the time from the start of the camera’s shooting to the capture of the structured light image of the line in units of s.

## 4. Algorithm for Image Segmentation of Adherent Peanut Based on Three-Dimensional Morphology

In order to solve the limitations of 3D morphology analysis under peanut adhesion, this study employs a roundabout strategy [25]. Firstly, the initial adherent 3D peanut model will be segmented using the region growing segmentation algorithm to separate individual peanuts in 3D morphology. Subsequently, the contour of the segmented individual peanut point cloud model will be extracted using the rolling ball method to generate a two-dimensional contour image. Finally, the contour image will serve as a template for achieving two-dimensional image segmentation through template multiscale searching and matching.

### 4.1. Point Cloud Segmentation Based on 3D Region Growth

**Figure 5:**
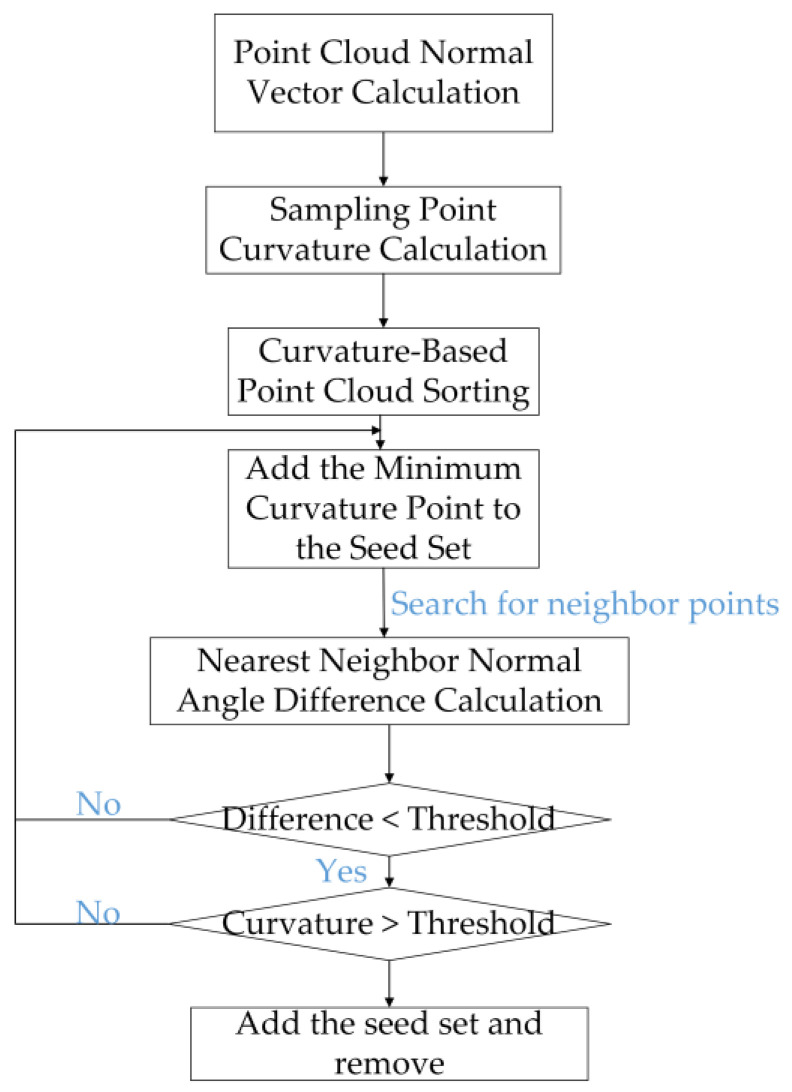
Three-dimensional (3D) regional growth algorithm flow.

The normal directions of the planes on the surface of the peanut point cloud differ greatly, while the normal directions of the adhering parts between the peanuts do not differ much. Therefore, the regional growth [26,27] method is used to segment the point cloud. The normal vector and the curvature are selected as the region growth features. The specific process is shown in Figure 5.

In this paper, a local surface-fitting method is used to compute the normal vector of a point cloud. The computation of the normal vector of a target point is approximated as the computation of the normal vector of a local tangent plane passing through the point. The normal vectors are obtained by least-squares plane fitting to the target point and the sampled points in its surrounding domain. The normal vectors of the fitted plane are basically perpendicular to the direction of the line connecting the target point and each of its nearest neighbor points. Assuming that the number of nearest neighbor points is k, each spatial point is denoted as ${{[X}_{1},Y_{2},Z_{3}]}^{T}$, and all the nearest neighbor point clouds are denoted as $P_{i}=[X_{1i},X_{2i},X_{3i}](i=1,2,\ldots ,k)$; the expression of the target function with the target point normal vector n as the parameter to be solved is as follows:

(5) $$F\left(n\right)=min\sum_{i=1}^{k}{\left({\left(X_{i}-\frac{1}{k}\sum_{i=1}^{k}X_{i}\right)}^{T}n\right)}^{2}(\left\|n\right\|=1)$$

Let $f\left(n\right)=min(n^{T}Sn)$, where $S=(X_{i}-\frac{1}{k}\sum_{i=1}^{k}X_{i}){\left(X_{i}-\frac{1}{k}\sum_{i=1}^{k}X_{i}\right)}^{T}$, $n^{T}n=1$, the constructed matrix S is the covariance matrix, and minimize the value of $f\left(n\right)$; then, we can see that the normal vector n is an eigenvector of the covariance matrix S, which can be obtained from the point cloud normal vector through principal component analysis.

After constructing the covariance matrix of the domain point cloud set, the curvature of the sampled points can be calculated based on the eigenvalues of the matrix at the same time. Assuming that the three eigenvalues of the matrix are $\lambda_{1},\lambda_{2},\lambda_{3}$, the curvature of the point ρ is calculated as follows:

(6) $$\rho =\frac{\lambda_{1}}{\lambda_{1}+\lambda_{2}+\lambda_{3}}$$

The next step involves performing region growth segmentation based on the specified normal vectors and curvature values.

1. Calculate the normal vector and curvature of each point in the point cloud set one by one;
2. Arrange the point cloud dataset according to the curvature value. Select the point with the lowest curvature as the initial seed point and add it to the seed point set;
3. Use the KD-Tree algorithm to search the k domain points of the selected seed point. Evaluate the angle between the normal vectors of the neighboring points and the current seed point one by one. If the angle is below a predefined threshold, assess whether the curvature of the neighboring point is less than a specified curvature threshold;
4. If a domain point satisfies both the normal pinch angle constraint and the curvature threshold limit, the point is added to the current set of seed points and removed from the original point cloud dataset. The segmentation of a peanut model is completed when there are no more points that meet these conditions in the remaining point cloud data;
5. Continuously loop the execution of steps (2)–(4) until all eligible point cloud data are effectively divided into a number of independent subsets of regions;
6. The algorithm terminates when the number of remaining point clouds is less than the set minimum number of segmentation region points.

Based on the above process, the segmentation can be realized for the 3D model of an adherent peanut.

### 4.2. Point Cloud Edge Contour Extraction

**Figure 6:**
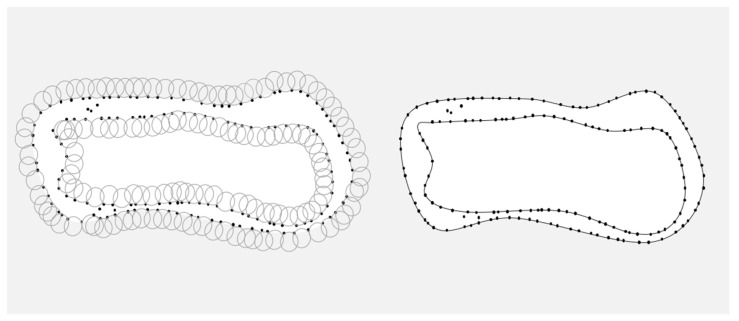
Boundary extraction by rolling ball method.

The edge contour lines of each peanut were extracted using the rolling ball method for matching the segmentation of 2D adhesion images, and Figure 6 shows the schematic diagram of the boundary extraction by the rolling ball method.

**Figure 7:**
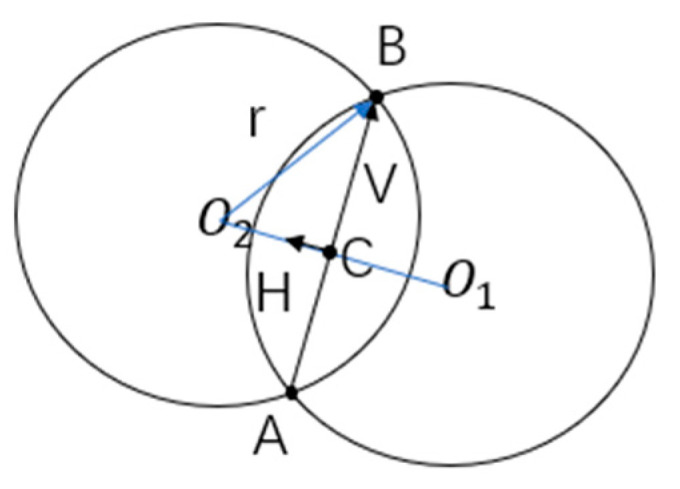
Geometric relationship diagram of the rolling ball method.

The specific process of the rolling ball method [28] is as follows:

1. Initialize the circle radius parameter of the roll method, traversing every AB line segment in the computational point cloud;
2. If the length of AB is greater than the diameter, it is considered to be outside the valid search area;
3. Based on the geometric principles shown in Figure 7, calculate the center of the circle $O_{1}$ and $O_{2}$;
4. Determine the direction vector V of the line segment AB, solve for the coordinates of the center of AB at C, and compute the perpendicular vector H of V;
5. Calculate the chord length AB of L, and solve for the perpendicular distance D from the center of the circle $O_{3}$ to the line segment AB according to the formula for the center of the circle $O_{3}=C\pm D*H$;
6. If there is a point in the interior of either circle that is not included in the point set Z, then A,B are considered as boundary points;
7. After completing the above steps, the contour boundary information of the point cloud data has been successfully extracted.

### 4.3. Image Adhesion Segmentation Based on Template Matching

**Figure 8:**
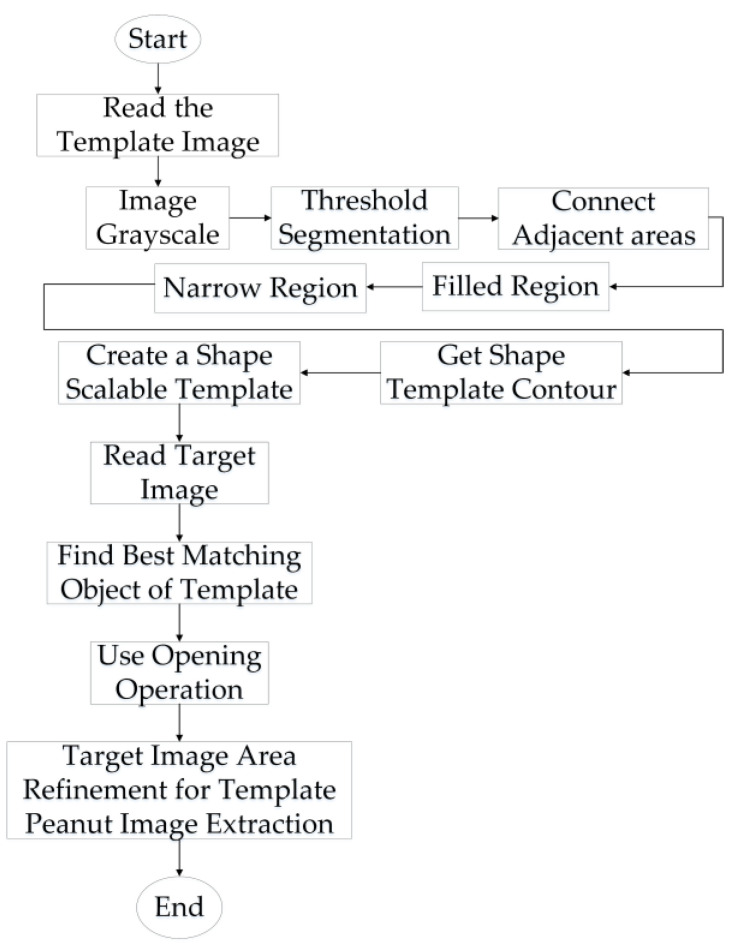
Matching-based segmentation algorithm flow.

After extracting the edge contours, the resulting peanut contours are used for feature matching and segmentation of the adherent peanut image. Feature-based matching is the process of matching images by establishing correspondence between the feature points in two images. Due to the low number of image feature points and their high sensitivity to positional transformations, both the amount of computational load in the matching process can be greatly reduced, and the accuracy of the matching can be improved [29]. The specific process is shown in Figure 8.

First, the 2D contour template image of the peanut is read. The image is then converted to gray-scale, undergoes threshold segmentation, connects adjacent areas, fills areas, segments the image based on the area, extracts the outline, and creates a shape-scalable template. Once the template is established, the target peanut adhesion image can be matched and segmented. To match the image, the optimal position for matching the peanut template is explored from top to down at the appropriate image pyramid level, utilizing a multiscale search. Meanwhile, the minimum distance accuracy threshold in the search process is determined using the least-squares method. By minimizing the distance between the model point and its corresponding point in the image, the line segment between the template point and the image point is defined by the tangent line between the points. Subsequently, the sub-pixel accuracy of the template is achieved by considering parameters such as the rotation angle, scaling ratio, and translation distance, thereby effectively enhancing the matching accuracy [30]. Assuming the image edge point is $S_{i}={\left(S_{ix},S_{iy},1\right)}^{T}$, the template feature point is $S_{i}={\left(D_{ix},D_{iy},1\right)}^{T}$, and the template point normal vector is $N_{i}={\left(N_{ix},N_{iy},1\right)}^{T}$, the rigid body transformation matrix is

(7) $$W=\left(\begin{array}{ccc} \cos\theta & -\sin\theta & x\\ \sin\theta & \cos\theta & y\\ 0 & 0 & 1 \end{array}\right)\approx \left(\begin{array}{ccc} 1 & -\theta & x\\ \theta & 1 & y\\ 0 & 0 & 1 \end{array}\right)$$

The minimization distance formula is as follows:

(8) $$\begin{array}{c} F\left(l\right)=\left(W\cdot S_{i}-D_{i}\right)\cdot N_{i}=\left(\left(\begin{array}{ccc} 1 & -\theta & x\\ \theta & 1 & y\\ 0 & 0 & 1 \end{array}\right)\cdot \left(\begin{array}{c} S_{ix}\\ S_{iy}\\ 1 \end{array}\right)-\left(\begin{array}{c} D_{ix}\\ D_{iy}\\ 1 \end{array}\right)\right)\cdot \left(\begin{array}{c} N_{ix}\\ N_{iy}\\ 1 \end{array}\right)\\ \;\;\;\;\;\;\;\;\;\;\;\;=N_{iy}x+N_{iy}y+\left(S_{ix}N_{iy}-S_{iy}N_{ix}\right)\theta +\left(S_{ix}-D_{ix}\right)N_{ix}+(S_{iy}-D_{iy})N_{iy} \end{array}$$

**Figure 9:**
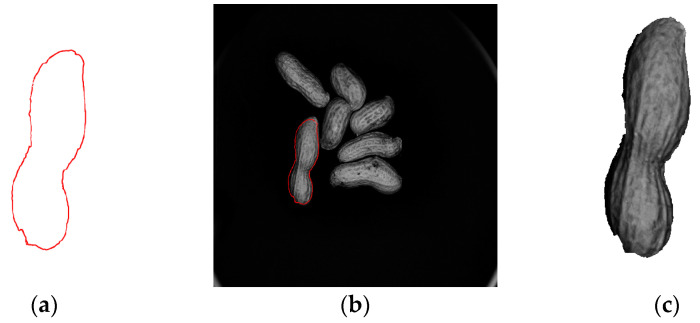
Peanut template-matching segmentation effect: (**a**) peanut contour template; (**b**) matching result; (**c**) segmentation result.

Once the template-matching process is successfully completed, the next step is to extract the target region from the original image that accurately matches the template. This region contains a single, complete peanut image that can be successfully segmented. The experimental process is shown in Figure 9.

As shown in Figure 9, the template contour obtained through the rolling ball method aligns closely with the peanuts in the target peanut image, displaying appropriate proportions. This outcome validates the rationality and precision of the three-dimensional reconstruction using the line structured light system. It also shows that the algorithm can achieve successful matching and the precise segmentation of peanuts.

**Figure 10:**
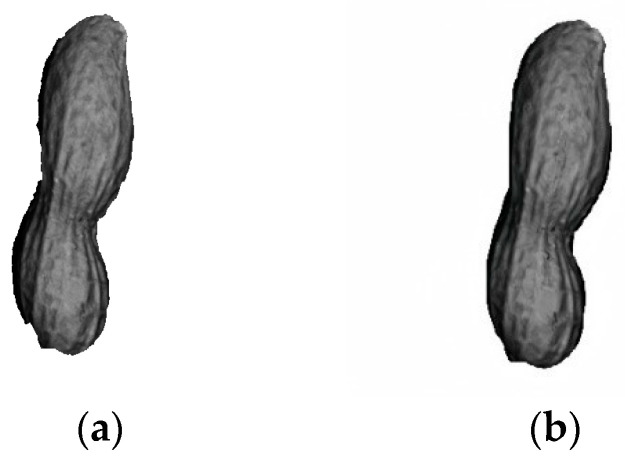
Comparison of opening operation: (**a**) before the opening operation; (**b**) after the opening operation.

During the three-dimensional segmentation of the peanut point cloud and the subsequent edge contour extraction process, issues such as partial missing edges, concave edges, and sharp corners may arise, leading to an incomplete and non-smooth contour. To ensure the integrity of the peanut shape and facilitate the effective extraction of peanut features for quality inspection, this study proposes repairing the matched peanut target region using the opening operation [31]. This process aims to enhance the smoothness and completeness of the peanut’s contour and eliminate fine burrs. The effect is shown in Figure 10.

## 5. Experimental Results and Analysis

The “Zhejiang Xinchang Xiaojingsheng” peanut is selected as the test object to validate the method proposed in this paper. The camera is a MV-CA050-10GMGC HIKVISON industrial camera, manufactured by Hangzhou Hikvision Digital Technology Co., Ltd., located in Hangzhou, China. The wavelength of the red laser is 650 nm, and the power is 100 mW.

The internal reference matrix for the camera calibration is

$$M=\left(\begin{array}{ccc} 10275 & 0 & 1224\\ 0 & 10272 & 972\\ 0 & 0 & 1 \end{array}\right)$$

The rotation matrix is

$$R=\left(\begin{array}{ccc} -0.7651 & -0.6438 & -0.0116\\ 0.6439 & -0.7651 & -0.0003\\ -0.0087 & -0.0077 & 0.9999 \end{array}\right)$$

The translation matrix is

$$T=\left(\begin{array}{c} 30.3430\\ 12.0417\\ 533.1570 \end{array}\right)$$

The coefficients of the plane equation after calibration of the optical plane of the line structure are

a=0.000022,b=−0.000003,c=0.001877,d=1

**Figure 11:**
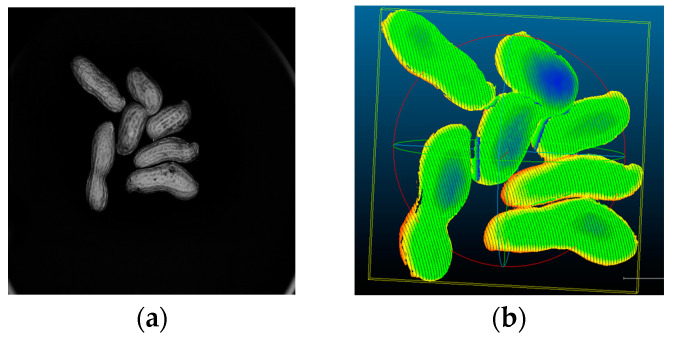
Peanut 3D reconstruction effect: (**a**) peanut 2D original; (**b**) peanut 3D point cloud.

The peanuts will be randomly placed on the motion guide platform. Each experimental image acquisition will contain three to eight peanuts. Initially, we set the guide rail movement speed to 0.1 mm/s and determined the movement distance to ensure that the first peanut entered the camera’s field of view and the last peanut disappeared from the camera’s field of view. The industrial cameras were configured to automatically capture one image per second to accurately capture images of laser streaks shining on peanuts at different translation positions. The center line of each laser stripe image is extracted, and the three-dimensional point cloud data of each image are calculated using the correspondence between the pixel coordinate system and the world coordinate system. The images are taken in sequence to calculate the position of each peanut relative to the initial position of the scanning distance d, enabling the reconstruction of the laser center line by sequentially stitching images in the same direction as the guide rail movement. This process results in the complete three-dimensional outline of the peanuts, ensuring that the point cloud model accurately represents the actual size of peanuts. The three-dimensional reconstruction of the peanuts is illustrated in Figure 11.

As shown in Figure 11, the three-dimensional model of a peanut reconstructed by a line structured light system exhibits high integrity and morphological similarity. Nevertheless, the figure also reveals the presence of a certain number of redundant point cloud stray points between individual adherent peanuts, which necessitate removal through three-dimensional segmentation.

**Figure 12:**
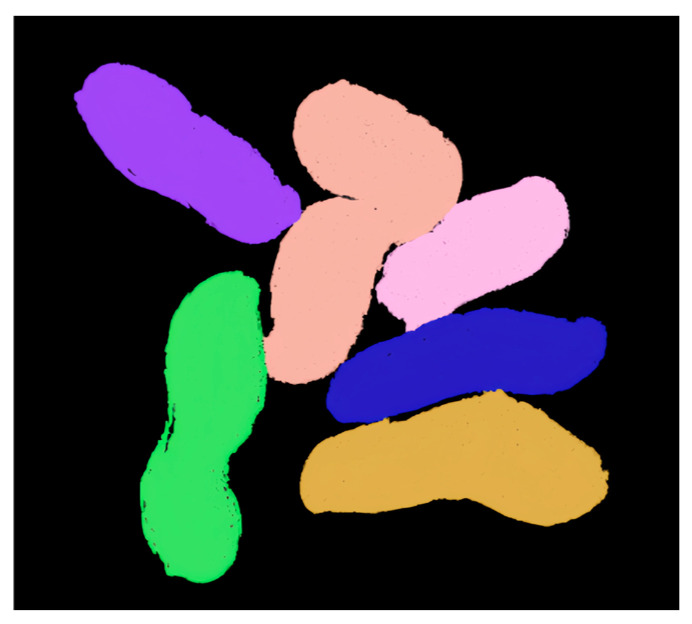
Adhesive peanut 3D segmentation.

Since the 3D reconstructed peanut model contains background, the peanut point cloud model is preprocessed based on the elevation interval to exclude points outside the threshold range. Subsequently, 3D segmentation is performed, and the segmentation results are shown in Figure 12. The region growing algorithm can effectively segment the adhesion state between the three-dimensional peanut models, maintaining the same number of peanuts before and after segmentation. The individual peanuts are fully intact with no missing or falsely segmented parts.

**Figure 13:**
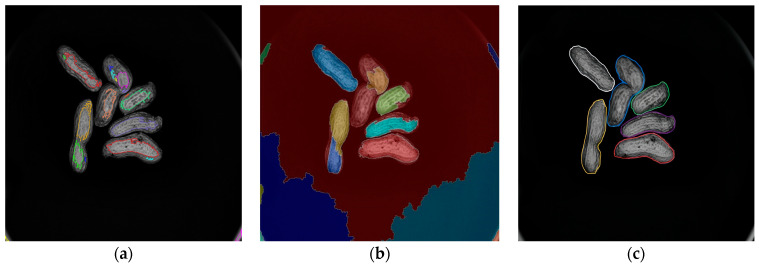
Segmentation results of different algorithms: (**a**) watershed algorithm based on distance transformation; (**b**) watershed algorithm based on gradient transformation; (**c**) proposed algorithm.

The distance transform-based watershed algorithm [32], the gradient transform-based watershed algorithm [33], and the algorithm proposed in this paper are used to segment the peanut adhesion image, respectively. The experimental results are shown in Figure 13.

In Figure 13a, the watershed algorithm based on distance transform cannot completely segment the adhesive peanuts, especially those with complex shapes such as axe-shaped and wasp waist peanuts, similar to ordinary peanuts. There is a phenomenon of under-segmentation. In Figure 13b, the watershed algorithm based on gradient change also shows the phenomenon of over-segmentation of complex peanut morphology, with less under-segmentation compared to (a), but it still exists. Figure 13c shows the adhesion segmentation results using the proposed algorithm. The adhesive peanuts are effectively segmented. Each peanut is accurately segmented with no over-segmentation or under-segmentation.

In order to evaluate the effectiveness of the segmentation algorithm more clearly, the segmentation accuracy was used as a key performance parameter to measure the effectiveness of the segmentation of adherent peanuts. The accuracy rate is defined as

(9) $$P=\frac{K_{1}}{K_{2}}\times 100\%$$

where $K_{1}$ is the number of pixels of a single peanut after matching the segmentation algorithm, and $K_{2}$ is the number of complete pixels of a single peanut.

In order to determine the total number of pixels in a single peanut, one image acquisition is conducted for each peanut separately, and the accuracy results are shown in Table 1.

As can be seen from Table 1, the average segmentation accuracy of the algorithm in this paper is the highest, the running time of the algorithm is moderate, and the segmentation effect is the best. It shows that the algorithm proposed in this paper is more suitable for the segmentation of adhesion images of peanuts with non-full convexity, complex surface texture, and diverse structures. However, when applied to simple and regular objects such as beans and rice grains, the algorithm in this paper is too complicated, and the relative efficiency is reduced compared with other adhesion segmentation algorithms such as the watershed algorithm.

In order to verify the accuracy and reliability of the proposed algorithm, 400 peanut samples with different shapes and sizes were used for quantitative experiments. The average successful segmentation rate is used as an evaluation index to analyze the influence of various factors on the segmentation results. It is assumed that the number of peanuts in each experimental group is m, and the number of pixels in the segmented region after each peanut is segmented by the algorithm in this paper is $z_{i}(i=1,2,\ldots ,m)$, corresponding to the complete pixel number is $Z_{i}(i=1,2,\ldots ,m)$. The formula for the average successful segmentation rate is as follows:

(10) $$\sigma =\frac{\sum_{i=1}^{m}z_{i}}{\sum_{i=1}^{m}Z_{i}}\times 100\%$$

i. Effect of light source intensity on edge position of 2D target image

**Figure 14:**
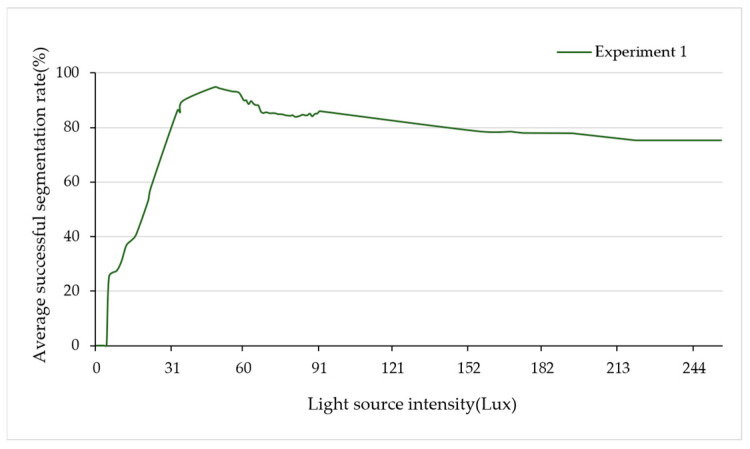
Adhesion segmentation experiment of different light source intensities.

The change in light source intensity affects the gray value and gray distribution of pixels at the edge position. In high-light or low-light environments, the accuracy of the template-matching algorithm in identifying edges decreases, impacting the precise matching and extraction of the peanut contour, and ultimately affecting the segmentation accuracy. To investigate the impact of light source intensity changes on accuracy, foreground light illumination was chosen as the illumination mode. A fixed set of experimental samples served as the benchmark, and the illumination intensity was gradually adjusted using the light source controller. The successive collection of 2D target images for segmentation was conducted. Figure 14 shows the experimental results based on the variation in light source intensity.

As can be seen from Figure 14, when the intensity of the light source is 50 Lux, the average successful segmentation rate is the best. With an increase in the intensity of the light source, the segmentation rate decreases. Excessive light intensity can lead to pixel saturation, a distortion of image details, and a shift in edge position positions toward size reduction, ultimately impacting segmentation results. This highlights the importance of setting the light source intensity appropriately to ensure a high segmentation success rate in practical applications.

ii. Effect of different adhesion states on adhesion segmentation experiment

**Figure 15:**
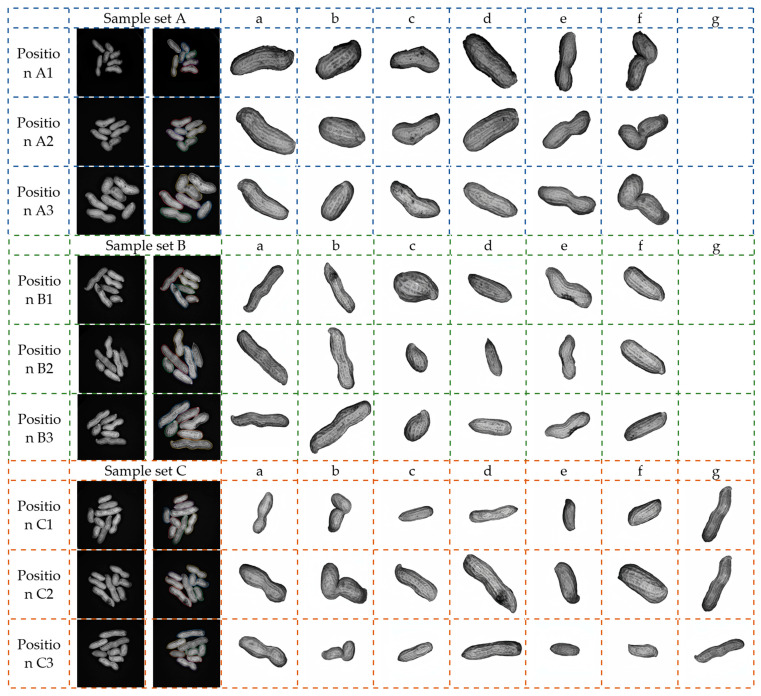
Segmentation results of different adhesion conditions.

In consideration of the impact of various adhesion states on the proposed algorithm, the identical group of sample sets underwent testing under various adhesion conditions while maintaining a constant light source intensity (50 Lux). Figure 15 presents the segmentation results for three sample sets. It is evident that the proposed algorithm effectively segments the peanuts across different adhesion conditions within the same sample set.

From Figure 15, it can be seen that the algorithm proposed in this paper has achieved good segmentation results for peanuts in nine groups of experiments across three sample sets. Each sample set was divided into three different adhesion conditions. The first column image in each experiment represents the original target adhesion image, the second column image shows the segmentation result of the proposed algorithm, and the subsequent columns a–g show the effect of each peanut segmentation. Regardless of the increase or decrease in the number of peanuts or the diversity of the adhesion state, including the similarities and differences in the morphology of the peanut samples themselves (such as the axe-shaped peanut f in sample set A, the lollipop peanuts a and b with longer pods in sample set B, and the cocoon peanuts e and f with higher similarity in sample set C), the algorithm can consistently achieves accurate segmentation, demonstrating a high degree of robustness. The edge of the segmented peanut area is relatively smooth, with high integrity, and clear surface texture, which facilitates the subsequent extraction of peanut phenotypic parameters for easy classification. However, it is worth noting that the segmentation of peanut b in experiment A1 and peanut e in experiment B2 is partially incomplete due to the three-dimensional reconstruction model being affected by occlusion between peanuts, leading to incomplete contour integrity and ultimately affecting the segmentation results, which need further optimization.

iii. Effect of peanut effective ratio per unit area on adhesion segmentation experiment

To investigate the impact of the number of peanuts in the camera’s field of view on the segmentation effect, this study designed experiments under a light intensity of 50 Lux. The variable was the effective proportion of peanuts per unit area, and new peanut sample sets were selected for each experiment to ensure the diversity of the experimental subjects.

**Figure 16:**
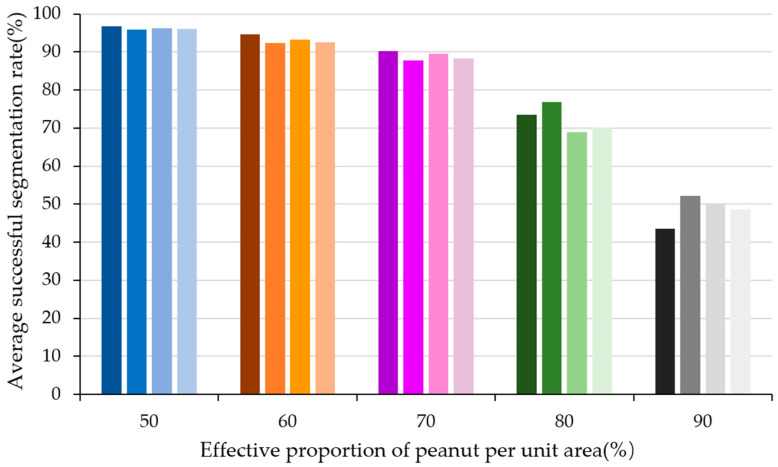
Peanut effective proportion adhesion segmentation experiment.

The experimental results are shown in Figure 16.

In Figure 16, each column of a different color represents a distinct set of experimental samples, and the proportion of peanuts in its unit area is equal to the corresponding horizontal coordinate number. It can be seen that when the peanut proportion is 50%, the algorithm achieves the best segmentation performance with an average successful segmentation rate above 95%. However, as the effective proportion of peanuts in the camera’s field of view increases, especially when the peanuts are tightly clustered and the proportion reaches 90%, the average successful segmentation rate significantly decreases, with over half of the experimental groups having a segmentation rate below 50%. Analysis indicates that when the proportion of peanuts in the camera’s field of view is high, meaning there are many peanuts that are closely clustered, the number of noise points generated by the line structured light system during the reconstruction of the 3D model increases, affecting the 3D segmentation effect and ultimately leading to a decrease in the average successful segmentation rate.

iv. Effect of different similar peanut quantity on adhesion segmentation experiment

**Figure 17:**
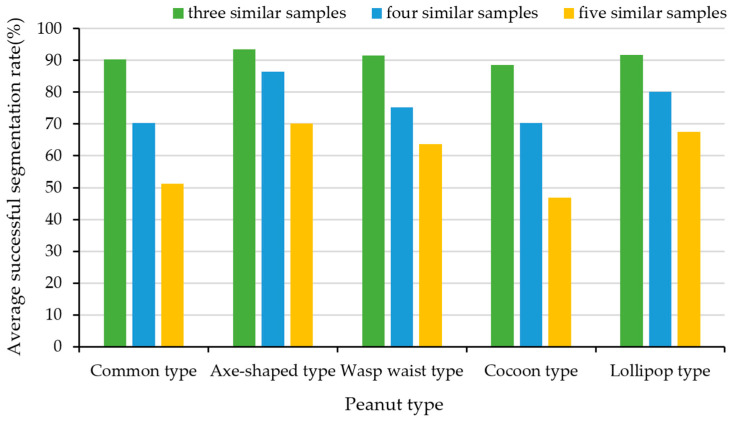
Adhesion segmentation experiment of different numbers of similar samples.

Under the conditions of a light intensity of 50 Lux and a 60% effective proportion of peanuts, this study designed an experiment to investigate the impact of the number of similar peanuts in the sample set on the adhesion segmentation algorithm. The experimental results for the common type, axe-shaped type, wasp-waist type, cocoon type, and lollipop-type peanuts are shown in Figure 17.

As shown in Figure 17, when there are only three similar peanuts in the experimental sample set, the proposed algorithm maintains a high average successful segmentation rate, which is mostly around 90%. However, as the number of similar peanuts increases, the accuracy of template matching significantly decreases, thereby affecting the final average successful segmentation rate. Moreover, compared to common-type and cocoon-type peanuts, which lack distinct waist features, the template-matching accuracy is lower for peanuts like axe-shaped-type, wasp-waist-type, and lollipop-type peanuts. This indicates that when the peanut template lacks prominent features, having too many similar peanuts can greatly impact the experimental segmentation results.

## 6. Conclusions

In this paper, a peanut adhesion segmentation algorithm based on multimodal fusion is proposed, which employs the line structured light technique for the three-dimensional reconstruction of adhesion peanuts, and recognizing the complexity of segmenting adhesion peanut images and the importance of accurate peanut quality detection, a 3D reconstruction–3D segmentation–2D inverse mapping segmentation strategy is proposed. The experimental results show that the 3D peanut model incorporating a high degree of information more easily realizes the adhesion segmentation of peanuts, and the inverse mapping of the 3D segmentation results onto the contour on the 2D plane not only ensures smooth segmentation edges but also significantly enhances the segmentation accuracy. This approach also preserves the surface features of the peanut shell, which provides an effective guarantee for the extraction of the shape parameters of the peanut at a later stage. However, during the 3D reconstruction of peanuts, the height differences and mutual occlusion between individual peanuts lead to incomplete point cloud models, affecting the precision of segmentation. Additionally, when the number of adhered peanuts is high, the generated point cloud model contains many noise points, which also impacts segmentation accuracy. Subsequent morphological opening operations cannot completely eliminate the burrs at the peanut edges. Therefore, future work will focus on optimizing the line structured light scanning system to reduce reconstruction errors, further enhancing the overall performance of the segmentation algorithm. Ongoing efforts will aim to achieve more comprehensive and smoother segmentation results, thereby enabling increased levels of automation and accuracy in peanut image segmentation. This advancement will offer significant benefits over traditional peanut quality classification projects that rely on manual segmentation and annotation.

## Figures and Tables

**Table 1:** Comparison of different segmentation algorithms.

Peanut Sample	Acc of Watershed Algorithm Based on Distance (%)	Acc of Watershed Algorithm Based on Gradient (%)	Acc of Proposed Algorithm (%)	
			Pre-Opening	Post-Opening
a	76.9	70.8	91.2	95.9
b	60.3	73.7	93.8	96.7
c	81.4	82.6	93.5	97.4
d	66.5	88.2	91.6	96.6
e	51.8	65.5	93.4	96.1
f	37.2	41.3	97.5	98.3
Average Acc(%)	62.3	70.35	93.5	96.8
Runtime/s	1.03	3.24	1.58	

## Data Availability

Data are contained within the article.
